# The Association Between Hormonal Contraceptives and Lower Extremity Injuries in Female Athletes With Sports-Related Clinical Encounters

**DOI:** 10.1177/19417381261479648

**Published:** 2026-09-11

**Authors:** Ahad A. Kesaria, Alice Lee, Alexander Goodsett, Margaret A. Goodwin, William M. Weiss

**Affiliations:** † John Sealy School of Medicine, The University of Texas Medical Branch, Galveston, Texas; ‡ Department of Orthopaedic Surgery and Rehabilitation, The University of Texas Medical Branch, Galveston, Texas

**Keywords:** ankle/foot, athletic training, injuries/conditions related to specific sports, medical aspects of sports, stress fractures

## Abstract

**Background:**

Hormonal fluctuations may influence musculoskeletal stability in women, raising interest in whether hormonal modulation through contraceptive use alters susceptibility to lower-extremity injuries in sports-related settings.

**Hypothesis:**

Systemic hormonal contraceptive use will be associated with lower rates of common lower-extremity injuries among female athletes with sports-related clinical encounters.

**Study Design:**

Cohort study.

**Level of Evidence:**

Level 3.

**Methods:**

Data were obtained using the TriNetX United States Collaborative Network. Female athletes aged 18 to 65 years who had encounters documented in sports or athletic environments were identified within a 20-year period. Cohort A included athletes with documented systemic hormonal contraceptive use while cohort B included athletes without contraceptive exposure. A 1:1 propensity score matching was used to balance the cohorts for demographic and medical factors such as hypertension, type 2 diabetes, and obesity. Outcomes were evaluated over 5 years with injuries that included anterior cruciate ligament (ACL) sprain, meniscal tear, ankle sprain, patellofemoral disorders, lower extremity stress fractures, Achilles tendinopathy, iliotibial band syndrome, or greater trochanteric pain syndrome. Chi-square analysis, risk ratios (RRs), 95% CIs, and *P* values were calculated to compare outcomes, with significance at *P* < 0.05.

**Results:**

A total of 8783 contraceptive users were matched to 32,499 nonusers, which resulted in 8358 patients in each balanced cohort. In 5 years, contraceptive users demonstrated significantly lower risks of ACL sprain (1.7% vs 2.2%; RR 0.78, 95% CI 0.62-0.97; *P* = 0.03), meniscal tear (2.0% vs 2.5%; RR 0.78, 95% CI 0.64-0.97; *P* = 0.02), and stress fractures (0.2% vs 0.4%; RR 0.52, 95% CI 0.29-0.93; *P* = 0.02).

**Conclusion:**

Among women with sports-related encounters, systemic hormonal contraceptive use was associated with lower risk of ACL sprains, meniscal tears, and lower extremity stress fractures.

**Clinical Relevance:**

Hormonal modulation through contraceptive use may play a protective role in ligamentous and bony injury risk in female athletes.

Oral contraceptives (OCPs) are the form of birth control used most commonly in both the general population and female athletes today.^8^ While OCPs are used predominantly for their contraceptive purposes by athletes, other benefits include improved bone health and control of the menstrual cycle and premenstrual symptoms.^4^ Multiple formulations exist, comprised of estrogen and progesterone or progesterone only. In general, the administration of these exogenous hormones provides a negative feedback loop in the hypothalamic-pituitary-ovarian axis. This in turn inhibits pituitary production of follicle-stimulating hormone (FSH) and luteinizing hormone (LH), ultimately preventing ovulation as well as development of the ovarian follicle and corpus luteum.^19,21,24^ This mechanism of action has also been shown to decrease levels of relaxin,^12,18^ a hormone normally produced by the corpus luteum and uterus in nonpregnant women.^3,7,20,23^

Previous studies have indicated that OCPs have far reaching effects on a woman’s metabolism, body composition, and endogenous hormones. These may translate to changes in her physical performance, as well as musculoskeletal (MSK) stability.^9,29^ Subjectively, a woman may experience little to no difference, but the improvement of clinical premenstrual symptoms, such as fluid retention and weight gain, could help in reducing rates of MSK injuries.^22^

At the molecular level, multiple MSK tissues undergo different processes in direct response to the hormones affected by OCPs. These include most soft tissues stabilizers about the knee and ankle including the anterior cruciate ligament (ACL). In particular, ACL tears have been associated with increased levels of estrogen and relaxin - hormones that correlate with increased pelvic and peripheral joint laxity and may be involved in catabolic and/or inhibitory effects on fibrocartilaginous tissues and collagen in articular cartilage.^12,23^ However, Hashem et al^17^ also showed that estrogen and relaxin did not induce a significant change in the knee meniscal tissues of their animal model and that progesterone minimized these catabolic effects. The catabolic effects of estrogen on collagen have been shown in human models and are not limited to tendons; additional research has shown significant influence on tissues of the lower extremities due to the presence of estrogen receptors, such as the patellar tendon, the vastus lateralis muscles,^16^ and the Achilles tendon.^6^

Despite the large body of literature regarding the effects of OCPs on the MSK system, many of the results are conflicting. Given the increasingly growing number of female athletes using OCPs for contraception or any secondary reason, it is imperative to gain a better understanding of these relationships in a broader, clinical sense. This may serve to help female athletes make more informed choices in regards to their risks of sports injury. Here, we aim to evaluate the association between hormonal OCP use and lower extremity injuries in female adult athletes. We hypothesize that systemic hormonal contraceptive use will be associated with lower rates of common lower-extremity injuries among female athletes with sports-related clinical encounters.

## Methods

This retrospective cohort study was conducted using TriNetX, a United-States-based database that aggregates deidentified electronic health data from participating healthcare organizations (HCOs), including academic centers, hospitals, and private healthcare systems. The present analysis used data from the United States Collaborative network, including 68 HCOs. Because all data in TriNetX are deidentified, institutional review board approval and individual patient consent were not required for this analysis. The analysis included patients from November 17, 2005 to November 17, 2025.

Female patients aged 18 to 65 years with clinical encounters occurring in sports and athletics settings were identified using the International Classification of Diseases, Tenth Revision (ICD-10) code Y92.3 (“sports and athletics area as the place of occurrence of the external cause”). This code reflects the documented location or setting of the encounter rather than confirmed competitive athletic participation. Patients were stratified into 2 cohorts based on documented exposure to systemic hormonal contraceptives. Cohort A included patients with documented use of systemic hormonal contraceptives, identified using the National Library of Medicine Anatomical Therapeutic Chemical (ATC) classification code G03A. No minimum duration cutoff for systemic hormonal contraceptive exposure was applied. Cohort B included patients without documented exposure to systemic hormonal contraceptives. For each patient, the index date was defined as the first encounter at which cohort inclusion criteria were met.

To balance differences between cohorts, 1:1 propensity score matching (PSM) was performed using the TriNetX built-in propensity matching function. Variables matched for included age at index, race, ethnicity, and clinical comorbidities, including overweight and obesity, type 2 diabetes mellitus, essential hypertension, nicotine dependence, alcohol-related disorders, opioid-related disorders, and osteoarthritis of the knee. Available laboratory values, including body mass index (BMI) and hemoglobin A1c (HbA1c), were also included in the matching process (Table 1).

**Table 1.** Demographics of patients with systemic hormonal contraceptive use (Cohort A) versus patients without systemic hormonal contraceptive use (Cohort B) before and after 1:1 PSM

		Before PSM				
Cohort	Demographic	Mean ± SD	Patients, n	Percentage of cohort	*P*	SMD
A	Age at index, years	21.8 ± 9.0	8783	100	<0.001	0.199
B		19.6 ± 12.6	32,467	100		
A	Male, N		0	0	-	-
B			0	0		
A	Female, N		8783	100	-	-
B			32,467	100		
A	White, N		6336	72.1	<0.001	0.126
B			21,544	66.4		
A	African American, N		898	10.2	0.02	0.028
B			3599	11.1		
A	Asian, N		393	4.5	<0.001	0.077
B			2014	6.2		
A	American Indian, N		83	0.9	0.006	0.031
B			216	0.7		
A	Pacific Islander, N		239	2.7	0.52	0.008
B			57	0.4		
A	Other race, N		337	3.8	<0.001	0.074
B			1752	5.4		
A	Unknown race, N		497	5.7	<0.001	0.082
B			2499	7.7		
A	Not Hispanic or Latino, N		6795	77.4	<0.001	0.185
B			22,467	69.2		
A	Hispanic or Latino, N		1011	11.5	<0.001	0.146
B			5376	16.6		
A	Unknown ethnicity, N		977	11.1	<0.001	0.094
B			4624	14.2		
A	Overweight and obesity, N		1317	15	<0.001	0.369
B			1391	4.3		
A	Type 2 diabetes mellitus, N		246	2.8	<0.001	0.129
B			336	1		
A	Essential (primary) hypertension, N		493	5.6	<0.001	0.159
B			807	2.5		
A	Nicotine dependence, N		596	6.8	<0.001	0.202
B			828	2.6		
A	Alcohol-related disorders, N		194	2.2%	<0.001	0.140
B			187	0.6		
A	Opioid-related disorders, N		79	0.9	<0.001	0.090
B			73	0.2		
A	Osteoarthritis of the knee, N		119	1.4	<0.001	0.069
B			217	0.7		
A	HbA1c, mmol/mol	5.6 ± 1.2	1661	18.9	0.005	0.098
B		5.7 ± 1.2	1589	4.9		
A	BMI, kg/m^2^	26.6 ± 7.3	6749	76.8	<0.001	0.249
B		24.8 ± 6.9	10,271	31.6		
		After PSM				
Cohort	Demographic	Mean ± SD	Patients, n	Percentage of cohort	*P*	SMD
A	Age at index, years	21.7 ± 9.0	8358	100	<0.001	0.168
B		23.5 ± 12.4	8358	100		
A	Male, N		0	0	-	-
B			0	0		
A	Female, N		8358	100	-	-
B			8358	100		
A	White, N		6025	72.1	0.78	0.004
B			6009	71.9		
A	African American, N		855	10.2	0.98	<0.001
B			856	10.2		
A	Asian, N		378	4.5	0.11	0.025
B			422	5.0		
A	American Indian, N		74	0.9	0.61	0.008
B			68	0.8		
A	Pacific Islander, N		231	2.8	0.22	0.019
B			258	3.1		
A	Other race, N		324	3.9	0.87	0.002
B			320	3.8		
A	Unknown race, N		471	5.6	0.11	0.024
B			425	5.1		
A	Not Hispanic or Latino, N		6441	77.1	0.97	0.001
B			6439	77.0		
A	Hispanic or Latino, N		955	11.4	0.27	0.017
B			910	10.9		
A	Unknown ethnicity, N		962	11.5	0.26	0.017
B			1009	12.1		
A	Overweight and obesity, N		1033	12.4	0.74	0.005
B			1047	12.5		
A	Type 2 diabetes mellitus, N		212	2.5	0.29	0.016
B			234	2.8		
A	Essential (primary) hypertension, N		449	5.4	0.05	0.031
B			509	6.1		
A	Nicotine dependence, N		525	6.3	0.19	0.020
B			567	6.8		
A	Alcohol-related disorders, N		158	1.9	0.96	0.001
B			157	1.9		
A	Opioid-related disorders, N		57	0.7	>0.99	<0.001
B			57	0.7		
A	Osteoarthritis of knee, N		116	1.4	0.13	0.023
B			140	1.7		
A	HbA1c, mmol/mol	5.6 ± 1.2	1278	15.3	0.05	0.078
B		5.7 ± 1.1	1315	15.7		
A	BMI, kg/m^2^	26.2 ± 6.9	6324	75.7	0.08	0.031
B		26.0 ± 7.1	6370	76.2		

BMI, body mass index; HbA1c, hemoglobin A1c; PSM, propensity score matching; SMD, standardized mean difference.

Primary outcomes consisting of lower-extremity MSK injuries within 5 years of the index date as specified above were identified using ICD-10 diagnosis codes. The query was adjusted to ensure that this index date must have occurred before any documented outcome within the 5-year period. These included ACL sprain, meniscal tear, stress fractures of the tibia, fibula, ankle, foot, or toes, patellofemoral disorders, patellar subluxation or dislocation, Achilles tendinopathy, iliotibial band syndrome, greater trochanteric pain syndrome, and ankle sprain. The injury outcomes were selected before analysis based on commonly encountered lower-extremity MSK injuries in sports medicine. Each outcome was analyzed independently. Patients were counted once per outcome, although patients with multiple documented injuries could contribute to >1 outcome category. Statistical analyses were conducted in the TriNetX platform. Categorical outcomes were evaluated using chi-square testing, and standardized mean differences (SMDs) were used to assess covariate balance after PSM. Risk ratio (RR), absolute risk difference, and corresponding 95% CIs were calculated using TriNetX to compare outcomes between cohorts. Statistical significance was defined as a *P* value <0.05 (Table 2).

**Table 2.** Lower extremity MSK injury outcomes after 1:1 PSM of patients with systemic hormonal contraceptive use (Cohort A) versus patients without systemic hormonal contraceptive use (Control)

Outcome	Cohort A (N = 8358)	Cohort B (N = 8358)	Absolute risk difference, %	RR (95% CI)	*P* value
5-year outcomes					
ACL sprain	139 (1.7)	178 (2.2)	–0.5	0.78 (0.62-0.97)	0.03*
Meniscal tear	156 (2.0)	199 (2.5)	–0.5	0.78 (0.64-0.97)	0.02*
GTPS	226 (2.8)	210 (2.6)	0.2	1.1 (0.91-1.3)	0.36
Patellofemoral disorders	99 (1.2)	76 (0.9)	0.3	1.3 (0.99-1.8)	0.06
Achilles tendinopathy	45 (0.5)	36 (0.4)	0.1	1.3 (0.81-2.0)	0.31
Ankle sprain	282 (4.5)	342 (5.0)	–0.5	0.91 (0.78-1.1)	0.20
Subluxation and dislocation of patella	61 (0.7)	66 (0.8)	–0.1	0.93 (0.66-1.3)	0.69
Iliotibial band syndrome	28 (0.3)	26 (0.3)	0.0	1.1 (0.63-1.8)	0.78
Lower extremity stress fracture	17 (0.2)	33 (0.4)	–0.2	0.52 (0.29-0.93)	0.02

Categorical data presented as n (% of cohort). ACL, Anterior Cruciate Ligament; GTPS, greater trochanteric pain syndrome; MSK, musculoskeletal; PSM, propensity score matching; RR, risk ratio.

* *P* < 0.05.

## Results

A total of 41,282 female patients aged 18 to 65 years with encounters documented in sports and athletics settings were identified, including 8783 patients with documented systemic hormonal contraceptive use (Cohort A) and 32,499 patients without contraceptive exposure (Cohort B). After 1:1 PSM, 8358 patients remained in each cohort.

Differences in demographic characteristics and medical comorbidities between Cohort A and Cohort B were minimal based on chi-square testing after matching. Baseline balance was assessed using SMDs, with values <0.1 indicating appropriate matching across variables. The mean age was 21.7 years in Cohort A compared with 23.5 years in Cohort B. Although this age difference remained statistically significant, the SMD for age was <0.1, indicating acceptable balance after matching. Racial distributions were predominantly White in both cohorts, with 72.1% of Cohort A and 71.9% of Cohort B. Mean BMI was 26.2 kg/m^2^ and 26.0 kg/m^2^, and hbA1c levels were 5.6 mmol/mol and 5.7 mmol/mol in Cohorts A and B, respectively (Table 1).

**Figure 1.**
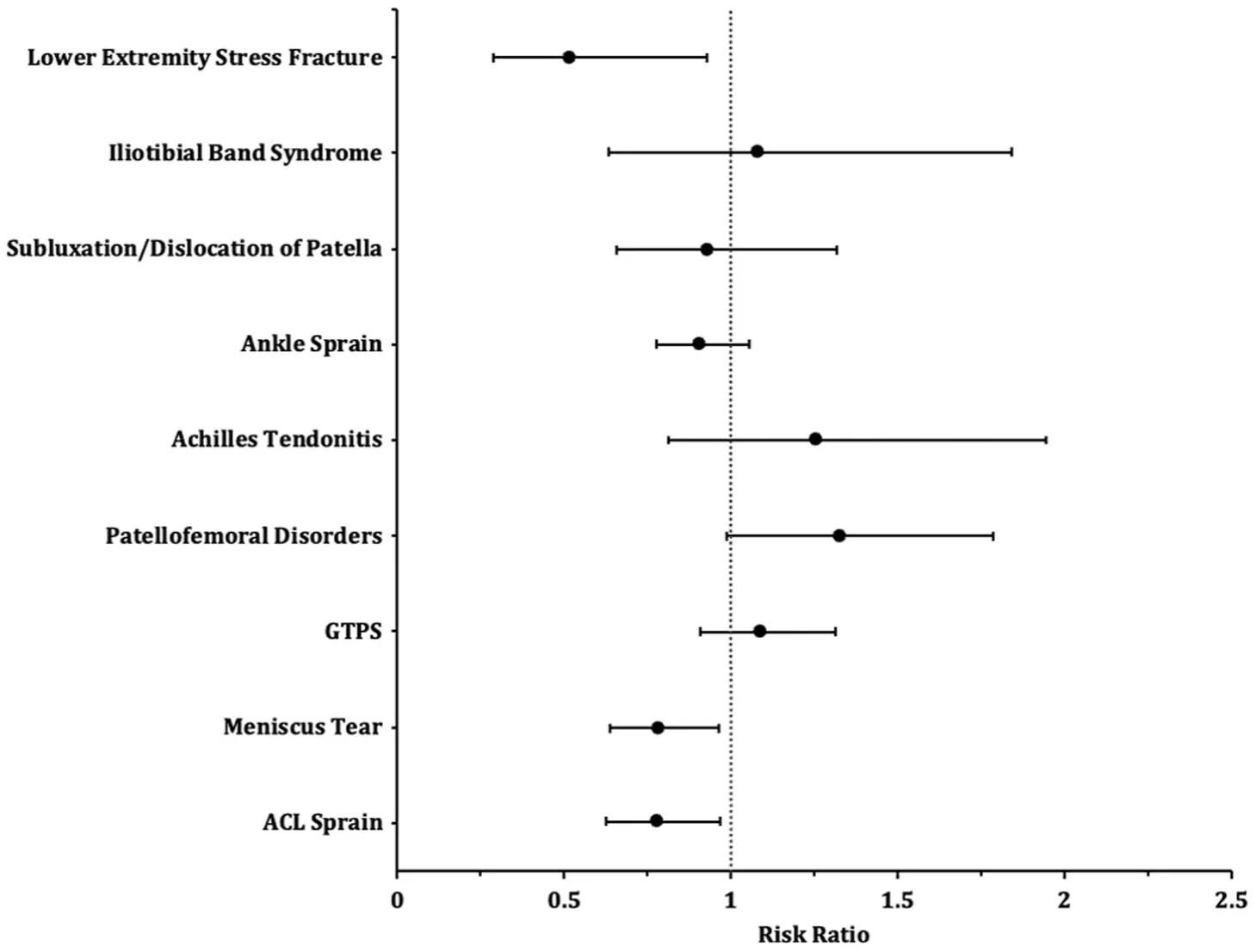
RRs of lower extremity injuries by systemic hormonal contraceptive use. ACL, anterior cruciate ligament; GTPS, greater trochanteric pain syndrome; RR, risk ratio.

Over the 5-year follow-up period, Cohort A demonstrated significantly lower proportions of ACL sprain compared with Cohort B (1.7% vs 2.2%; RR, 0.78; 95% CI, 0.62-0.97; *P* = 0.03). The proportion of meniscal tear was also reduced significantly among contraceptive users (2.0% vs 2.5%; RR, 0.78; 95% CI, 0.64-0.97; *P* = 0.02). In addition, lower extremity stress fractures occurred less frequently in Cohort A compared with Cohort B (0.2% vs 0.4%; RR, 0.52; 95% CI, 0.29-0.93; *P* = 0.02) (Figure 1, Table 2). Absolute risk differences for all outcomes are reported in Table 2.

No statistically significant differences were observed between cohorts for ankle sprain (0.034% vs 0.041%; RR, 0.91; 95% CI, 0.7-1.1; *P* = 0.20), patellar subluxation or dislocation (0.0073% vs 0.0079%; RR, 0.93; 95% CI, 0.66-1.3; *P* = 0.69), patellofemoral disorders (0.012% vs 0.0091%; RR, 1.3; 95% CI, 0.99-1.8; *P* = 0.06), iliotibial band syndrome (0.0034% vs 0.0031%; RR, 1.1; 95% CI, 0.63-1.8; *P* = 0.78), Achilles tendinopathy (0.0054% vs 0.0043%; RR, 1.3; 95% CI, 0.81-2.0; *P* = 0.31), or greater trochanteric pain syndrome (2.7% vs 0.025%; RR, 1.1; 95% CI, 0.91-1.3; *P* = 0.36) (Figure 1, Table 2).

## Discussion

Our results show that OCP use in female athletes aged 18 to 65 years is associated with lower risk of ACL sprains, meniscal tears, and stress fractures. Our findings regarding ACL sprains are consistent with other reported literature.^11,13,15,19,27^ For example, Dragoo et al^12^ found a significant relationship between increased serum relaxin concentrations in elite female athletes and the incidence of complete ACL tears.^9^ One of the hormonal effects caused directly by OCPs includes a decrease in relaxin concentrations. Fry et al^13^ showed a lower incidence of ACL injury requiring surgical reconstruction in people taking OCPs with the exception of 15- to 19-year-olds. While our study did not stratify by age, our results do show a more general association in a larger population size. Interestingly, our results show a significance with ACL sprains, rather than complete tears, which may point to a more moderate relationship between the effects of OCPs on the properties of the ACL. This may partially explain why many secondary reviews and meta-analyses fail to reliably show a relation between hormonal regulation in female athletes and the risk of ACL injury.^20,25^ In the present study, the ACL outcome was identified using ICD-10-CM coding for ACL sprain, which may include partial or complete ACL injuries. Because diagnosis codes do not reliably distinguish partial sprains from complete ruptures, our findings should be interpreted as an association with coded ACL sprain rather than surgically confirmed ACL rupture. Database studies, such as ours, can thus serve as a valuable tool in this growing body of research.

A similar protective effect of OCPs is seen regarding meniscal tears. For meniscal pathology, a direct hormonal explanation is not clear. The rabbit model studied by Hashem et al^17^ showed no significant change in meniscal collagen concentrations with exposure to varying combinations of estrogen, progesterone, and relaxin. This was in contrast to other tissues where an effect was seen such as the pubic symphysis, and articular cartilage.^17^ It is well established that meniscal tears are comorbid with ACL injuries.^1^ The protective effects of OCPs on the meniscus may instead be secondary to their primary effects on the ACL.

The effect of OCPs on GTPS is not well explored. While our study found no effect, other studies have found them to be harmful. For example, Smith et al^31^ found an increased association between progestin-only and combined-OCPs and GTPS and other hip pathologies. Given the relatively young mean age of the matched cohorts, interpretation of GTPS findings may be limited, as GTPS is described more commonly in older populations. As such, the effects of OCPs on soft tissue pathologies other than the ACL are unclear and remains an avenue of further investigation.

The impact of OCPs on stress fracture risk may be related to their modulation of bone mineral density and osteoporosis.^2,14^ While the relationship between stress fractures and athletics has been synthesized traditionally in what is known as the female athlete triad, consisting of osteoporosis, disordered eating, and amenorrhea, the International Olympic Committee has updated this to the syndrome of Relative Energy Deficiency in Sports (RED-S). This refers to impaired metabolic and endocrine functions caused by a relatively low energy availability.^26^ One of the physiologic functions that is disrupted in RED-S is bone metabolism, as seen in the increased risk of stress fractures in athletes with RED-S.^5^ Given our results showing a decreased association between OCPs and stress fractures, female athletes with RED-S may be a more targeted demographic to explore the clinical application of OCPs in the context of reducing the risk of lower extremity injuries. A randomized trial by Cobb et al^10^ showed that OCPs were associated with a 43% decrease in the rate of stress fractures among female runners. However, these results were limited by noncompliance, and findings were ultimately nonsignificant. In a different study, Cheng et al^9^ found an association between OCPs and decreased risk of stress fractures. Interestingly, this effect was seen specifically with OCPs and not with other forms of hormonal contraception such as intrauterine devices or injectables. Other authors such as Redinger and Baker^28^ and Ryan et al^30^ found no associations in prospective studies. Given the complex interplay between a patient’s age, hormonal status, dosing, composition, and even delivery, it is perhaps unsurprising that there are conflicting results. Based on this, we recommend that further clinical studies should be done to help clarify the individual impact of these variables.

Limitations of this study include the constraints seen in large database research and retrospective studies. The outcome diagnoses in TriNetX are defined and limited by the accuracy of the electronic health records maintained by the participating health institutions. Human error in the form of clerical mistakes or recall bias may introduce inaccuracies in the CPT codes utilized in this study. The Y92.3 code identifies encounters occurring in sports and athletics settings but does not confirm competitive athletic participation, sport type, level of play, activity exposure, or training volume. These factors are major determinants of lower-extremity injury risk and could not be assessed in this analysis. Because TriNetX does not reliably define injury mechanism, laterality, or concomitant injury patterns, we were unable to determine whether meniscal injuries occurred independently or in association with ACL injury. Although PSM was performed across multiple demographic, clinical, and laboratory variables, residual confounding from unmeasured factors remains possible. This may be more apparent when considering the high level of generalizability to our cohorts, wherein female athletes were not stratified by specific formulation and dosage of OCP, smaller age groups, and sport. The selected age range also excludes adolescent athletes and includes older adults who may be less likely to use hormonal contraceptives. Future studies with age-stratified analyses may better clarify whether associations differ across reproductive age groups. The use of ICD-10-CM coding introduces the potential for coding errors, misclassification, and incomplete capture of injuries. Hormonal contraceptives may be prescribed for both contraceptive and noncontraceptive indications, including menstrual regulation, dysmenorrhea, acne, and other medical indications. The specific indication for use was not evaluated in this analysis. Although outcomes were assessed after the index date and patients with previous documentation of each specific outcome were excluded from that outcome analysis, the initial reason for the sports-related encounter may have influenced subsequent injury risk. Finally, causality cannot be established.

## Conclusion

Among women with sports-related clinical encounters, systemic hormonal contraceptive use was associated with lower risk of ACL sprains, meniscal tears, and lower extremity stress fractures over a 5-year follow-up period. No statistically significant associations were observed for ankle sprain, patellar subluxation or dislocation, patellofemoral disorders, Achilles tendinopathy, iliotibial band syndrome, or GTPS. These findings suggest that hormonal contraceptive use may be associated with select lower-extremity injury patterns, although causal relationships cannot be determined from this retrospective database analysis. Future studies incorporating sport type, athletic exposure, contraceptive formulation, duration of use, menstrual history, and injury mechanism are needed to clarify these associations.

## References

[1] Adams BG Houston MN Cameron KL. The epidemiology of meniscus injury. Sports Med Arthrosc Rev. 2021;29(3):e24-e33. doi:10.1097/JSA.0000000000000329 34398119

[2] Ampatzis C Zervoudis S Iatrakis G Mastorakos G. Effect of oral contraceptives on bone mineral density. Acta Endocrinol Buchar. 2022;18(3):355-360. doi:10.4183/aeb.2022.355 36699167 PMC9867809

[3] Bathgate RAD Halls ML van der Westhuizen ET Callander GE Kocan M Summers RJ . Relaxin family peptides and their receptors. Physiol Rev. 2013;93(1):405-480. doi:10.1152/physrev.00001.2012 23303914

[4] Bennell K White S Crossley K. The oral contraceptive pill: a revolution for sportswomen? Br J Sports Med. 1999;33(4):231-238. doi:10.1136/bjsm.33.4.231 10450476 PMC1756183

[5] Brackel FN Munzinger R Bartosik M , et al. Impact of relative energy deficiency in sport (REDs) on bone health in elite athletes: a retrospective analysis. J Cachexia Sarcopenia Muscle. 2025;16(5):e70082. doi:10.1002/jcsm.70082 PMC12485273 41030229

[6] Bryant AL Clark RA Bartold S , et al. Effects of estrogen on the mechanical behavior of the human Achilles tendon in vivo. J Appl Physiol. 2008;105(4):1035-1043. doi:10.1152/japplphysiol.01281.2007 18566188

[7] Bryant GD. The detection of relaxin in porcine, ovine and human plasma by radioimmunoassay. Endocrinology. 1972;91(4):1113-1117. doi:10.1210/endo-91-4-1113 5065809

[8] Burrows M Peters CE. The influence of oral contraceptives on athletic performance in female athletes. Sports Med Auckl. 2007;37(7):557-574. doi:10.2165/00007256-200737070-00001 17595152

[9] Cheng J Santiago KA Abutalib Z , et al. Menstrual irregularity, hormonal contraceptive use, and bone stress injuries in collegiate female athletes in the United States. PM R. 2021;13(11):1207-1215. doi:10.1002/pmrj.12539 33340255 PMC8262270

[10] Cobb KL Bachrach LK Sowers M , et al. The effect of oral contraceptives on bone mass and stress fractures in female runners. Med Sci Sports Exerc. 2007;39(9):1464-1473. doi:10.1249/mss.0b013e318074e532 17805075

[11] DeFroda SF Bokshan SL Worobey S Ready L Daniels AH Owens BD. Oral contraceptives provide protection against anterior cruciate ligament tears: a national database study of 165,748 female patients. Phys Sportsmed. 2019;47(4):416-420. doi:10.1080/00913847.2019.1600334 30913940

[12] Dragoo JL Castillo TN Braun HJ Ridley BA Kennedy AC Golish SR. Prospective correlation between serum relaxin concentration and anterior cruciate ligament tears among elite collegiate female athletes. Am J Sports Med. 2011;39(10):2175-2180. doi:10.1177/0363546511413378 21737831

[13] Fry SA Hirpara A Whitney KE , et al. Use of hormonal contraceptives is associated with decreased incidence of anterior cruciate ligament injuries requiring reconstruction in female patients. Arthroscopy. 2025;41(9):3413-3421. doi:10.1016/j.arthro.2025.02.012 39983800

[14] Gambacciani M Monteleone P Ciaponi M Sacco A Genazzani AR. Effects of oral contraceptives on bone mineral density. Treat Endocrinol. 2004;3(3):191-196. doi:10.2165/00024677-200403030-00006 16026114

[15] Gray AM Gugala Z Baillargeon JG. Effects of oral contraceptive use on anterior cruciate ligament injury epidemiology. Med Sci Sports Exerc. 2016;48(4):648-654. doi:10.1249/MSS.0000000000000806 26540261

[16] Hansen M Miller BF Holm L , et al. Effect of administration of oral contraceptives in vivo on collagen synthesis in tendon and muscle connective tissue in young women. J Appl Physiol. 2009;106(4):1435-1443. doi:10.1152/japplphysiol.90933.2008 18845777

[17] Hashem G Zhang Q Hayami T Chen J Wang W Kapila S. Relaxin and β-estradiol modulate targeted matrix degradation in specific synovial joint fibrocartilages: progesterone prevents matrix loss. Arthritis Res Ther. 2006;8(4):R98. doi:10.1186/ar1978 PMC1779373 16784544

[18] Hatcher RA. Contraceptive Technology. 19th ed. Hartlepool: Ardent Media; 2007.

[19] Herzberg SD Motu’apuaka ML Lambert W Fu R Brady J Guise JM. The effect of menstrual cycle and contraceptives on ACL injuries and laxity: a systematic review and meta-analysis. Orthop J Sports Med. 2017;5(7):2325967117718781. doi:10.1177/2325967117718781 PMC5524267 28795075

[20] Khan-Dawood FS Goldsmith LT Weiss G Dawood MY. Human corpus luteum secretion of relaxin, oxytocin, and progesterone. J Clin Endocrinol Metabol. 1989;68(3):627-631. doi:10.1210/jcem-68-3-627 2918060

[21] Konopka JA Hsue LJ Dragoo JL. Effect of oral contraceptives on soft tissue injury risk, soft tissue laxity, and muscle strength: a systematic review of the literature. Orthop J Sports Med. 2019;7(3):2325967119831061. doi:10.1177/2325967119831061 PMC6431771 30923726

[22] Lebrun CM. Effect of the different phases of the menstrual cycle and oral contraceptives on athletic performance. Sports Med (Auckland NZ). 1993;16(6):400-430. doi:10.2165/00007256-199316060-00005 8303141

[23] MacLennan AH. Relaxin - a review. Aust N Z J Obstet Gynaecol. 1981;21(4):195-202. doi:10.1111/j.1479-828X.1981.tb00130.x 6280666

[24] Möller Nielsen J Hammar M . Sports injuries and oral contraceptive use: is there a relationship? Sports Med (Auckland NZ). 1991;12(3):152-160. doi:10.2165/00007256-199112030-00002 1784871

[25] Moriceau J Fevre A Domínguez-Balmaseda D González-de-la-Flor Á Simón-Areces J García-Pérez-de-Sevilla G. The influence of the menstrual cycle and oral contraceptives on knee laxity or anterior cruciate ligament injury risk: a systematic review. Appl Sci. 2022;12(24):12627. doi:10.3390/app122412627

[26] Mountjoy M Sundgot-Borgen JK Burke LM , et al. IOC consensus statement on relative energy deficiency in sport (RED-S): 2018 update. Br J Sports Med. 2018;52(11):687-697. doi:10.1136/bjsports-2018-099193 29773536

[27] Rahr-Wagner L Thillemann TM Mehnert F Pedersen AB Lind M. Is the use of oral contraceptives associated with operatively treated anterior cruciate ligament injury? A case-control study from the Danish knee ligament reconstruction registry. Am J Sports Med. 2014;42(12):2897-2905. doi:10.1177/0363546514557240 25428957

[28] Redinger AL Baker BS. Oral contraceptives and female rowers’ skeletal health. J Strength Cond Res. 2023;37(3):669-677. doi:10.1519/JSC.0000000000004308 36165993

[29] Rickenlund A Carlström K Ekblom B Brismar TB von Schoultz B Hirschberg AL. Effects of oral contraceptives on body composition and physical performance in female athletes. J Clin Endocrinol Metab. 2004;89(9):4364-4370. doi:10.1210/jc.2003-031334 15328063

[30] Ryan B Brezovec A Ying S Stahlman S Pier B. Contraceptive use and fracture risk in active-duty females - a case control study. Contracept Stoneham. 2026;153:111239. doi:10.1016/j.contraception.2025.111239 41027550

[31] Smith KL Shah AK Burkhart RJ , et al. Systemic hormonal contraception is associated with a greater rate of greater trochanteric pain syndrome, labral tear, and femoroacetabular impingement syndrome in female patients. Arthroscopy. 2025;41(12):5118-5124. doi:10.1016/j.arthro.2025.07.017 40769325

